# Preparation of Multiwalled Carbon Nanotubes/Hydroxyl-Terminated Silicone Oil Fiber and Its Application to Analysis of Crude Oils

**DOI:** 10.1155/2014/758043

**Published:** 2014-01-22

**Authors:** Shukui Zhu, Ting Tong, Wanfeng Zhang, Wei Dai, Sheng He, Zhenyang Chang, Xuanbo Gao

**Affiliations:** ^1^Key Laboratory of Tectonics and Petroleum Resources of Ministry of Education, China University of Geosciences, Wuhan 430074, China; ^2^Faculty of Earth Resources, China University of Geosciences, 388 Lumo Road, Wuhan 430074, China

## Abstract

A simple and efficient method to analyze the volatile and semivolatile organic compounds in crude oils has been developed based on direct immersion solid-phase microextraction coupled to comprehensive two-dimensional gas chromatography/time-of-flight mass spectrometry (DI-SPME-GC × GC/TOFMS). A novel fiber, multiwalled carbon nanotubes/hydroxyl-terminated silicone oil (MWNTs-TSO-OH), was prepared by sol-gel technology. Using standard solutions, the extraction conditions were optimized such as extraction mode, extraction temperature, extraction time, and salts effect. With the optimized conditions, a real crude oil sample was extracted and then analyzed in detail. It shows that the proposed method is very effective in simultaneously analyzing the normal and branched alkanes, cycloalkanes, aromatic hydrocarbons, and biomarkers of crude oil such as steranes and terpanes. Furthermore, the method showed good linearity (*r* > 0.999), precision (RSD < 8%), and detection limits ranging from 0.2 to 1.6 ng/L.

## 1. Introduction

As the major components in crude oils, volatile and semivolatile organic compounds have played important roles during petroleum exploration and development. Among the various classes of compounds in crude oil, some biomarkers such as terpanes and steranes are considered as the unique fingerprint of a specific oil and used to determine petroleum system characteristics such as origin, thermal maturity, and biodegradation level, as well as oil-oil and oil-source rock correlations [1]. At the same time, these molecules are also excellent indicators for tracing degree of weathering and the fate of spilled oil in the environment, due to their resistance to petrochemical and microbial degradation. Therefore, the qualitative and quantitative determination of volatile and semivolatile organic compounds in crude oil has attracted increasing attention.

Most existing analytical methods used to determine these organic compounds in crude oil are conventional one-dimensional gas chromatography coupled to mass spectrometry (1DGC/MS) or tandem mass spectrometry (GC/MS/MS) [2–4]. However, crude oils are complex matrices and consist of thousands of compounds, so the peak capacity of 1DGC is insufficient and peaks overlap seriously [5, 6]. In order to overcome these deficiencies, some labor-intensive and time-consuming sample preparation steps have to be used before GC analysis. The routine method is column chromatography on silica gel and alumina, by which crude oils are separated into different fractions such as saturated hydrocarbon fraction, aromatic fraction, and polar fraction [7]. The separated fractions are further concentrated and then injected to GC for analysis. During the sample preparation, large amounts of high-purity organic solvents that are potentially toxic and expensive are used. Additionally, manual concentration steps are usually conducted by vaporizing each collected fraction under nitrogen flow, which easily results in the loss of some light components. In order to obtain more accurate and efficient analysis of crude oils, some novel methods of sample pretreatment and chromatographic separation are quite required.

Solid-phase microextraction (SPME) is a simple, time-saving, solvent-free, low-cost, and efficient extraction technique, which integrates the extraction, preconcentration, and sample injection into one step [8]. It has gained widespread acceptance in the recent years and has been successfully applied to the extraction and enrichment of trace components in different sample matrices such as foods [9], environmental water or soils [10–12], and natural products [13, 14]. Sampling of analytes is done either through direct immersion of suitable fiber into a liquid phase (DI-SPME) or in headspace (HS-SPME), followed by thermal desorption of the extracted analytes in the hot injection port of GC [15]. However, as a promising method of sample pretreatment, only a few applications in the crude oils were reported and mainly focused on analysis of some special compounds or matrices, such as petroleum hydrocarbons in soils [16], methanol [17], and compounds with volatility less than n-Pentadecane (C_15_) [18] in the crude oils. Up to now, we are not aware of any report on the application of SPME in semivolatile compounds of crude oils, especially like petroleum biomarkers such as terpanes and steranes. The possible reason is as follows. Firstly, the maximum operating temperature of the existing commercial SPME fibers is no more than 280°C (except polyacrylate), which is relatively low when comparing with eluting temperature of these compounds. Secondly, the surface area or adsorption capacity of commercial fibers is low for complex composition of crude oil samples, which results in the low sensitivity of trace petroleum biomarkers. Thirdly, commercial SPME fibers are designed to extract either polar or nonpolar analytes from a given matrix, which is not suitable for crude oils comprising different chemical classes. Fourthly, thermal and solvent restrictions are encountered with traditional SPME fibers because the majority of these fibers are prepared by mere physical deposition of the polymer coating on the substrate of the fused-silica fiber [19].

In order to overcome the above shortages, a novel SPME coating made from multiwalled carbon nanotubes (MWNTs) and hydroxyl-terminated silicone oil (TSO-OH) was prepared by sol-gel technique in this study. Sol-gel technique can create surface-bonded SPME coatings with porous structure and good thermal stability. MWNTs have strong physical adsorption ability to hydrophobic organic compounds, good thermal, mechanical, and chemical stability, and high surface-to-volume ratio, and so forth. Based on the homemade SPME fiber, the volatile and semivolatile organic compounds in crude oils were extracted in DI-SPME mode and then analyzed by comprehensive two-dimensional gas chromatography coupled with time-of-flight mass spectrometry (GC × GC/TOFMS). The extraction temperature, extraction time, salts effect, volume of water added into the sample, and solvents addition were discussed in detail by using standard solutions. The individual components in crude oils were well separated and identified, such as normal and branched alkanes, acyclic isoprenoids, aromatic hydrocarbons, and polycyclic terpanes and steranes. Additionally, an accurate quantitative method was developed for the determination of some important petroleum biomarkers like polycyclic terpanes and steranes and so forth.

## 2. Experimental

### 2.1. Instrumentation

The GC × GC system consisted of a GC (7890A model, Agilent Technologies, Wilmington, DE, USA) equipped with a flame ionization detector (FID) and a time-of-flight mass spectrometer (Pegasus 4D, Leco Corp., St. Joseph, MI, USA). The dual-stage, quad-jet thermal modulator was used, and a detailed description of the modulator has been given in a previous publication [20]. The SPME devices were purchased from Supelco (Bellefonte, PA, USA).

DI-SPME-GC × GC/TOFMS analysis was performed using a DB-Petro column (50 m × 0.20 mm × 0.50 *μ*m) as the 1st column and a DB-17Ht column (1.5 m × 0.10 mm × 0.10 *μ*m) as the 2nd column, both of which were from J&W Scientific (Folsom, CA, USA). The carrier gas was helium (purity ≥ 99.9995%) with a flow rate of 1.0 mL/min. The injector temperature was 300°C. The 1st oven temperature program was as follows: 40°C for 1 min, 3°C/min to 300°C, hold for 30 min. The 2nd oven temperature was 10°C above the 1st oven. Modulation was carried out using a 15°C temperature offset and an 8 s modulation time (hot pulse 2 s). The mass spectrometer was operated at an acquisition rate of 100 spectra per second for a mass range of 50 to 550 u, using 70 eV electron impact ionization and 1500 V multichannel plate voltage. The ion-source temperature was 220°C and the transfer-line temperature was 300°C. The pressure inside the flight tube was 1.1 × 10^−7^ Torr.

To mix various solution ingredients thoroughly, an Ultrasonicator model KQ-50DE (Kunsan Ultrasonicator Instrument Corporation, Kunsan, China) was used. A centrifuge model TGL-16C (Shanghai Anting Instrument Factory, Shanghai, China) was used to separate the sol solution from the precipitate during fiber preparation. A magnetic stirrer DF-101B (Leqing, China) was used for stirring the sample during extraction.

### 2.2. Reagents and Materials

The MWNTs with purity 95% were purchased from Shenzhen Nanotech Port (Shenzhen, China). TSO-OH was purchased from Aldrich (Allentown, PA, USA). Tetraethoxysilane (TEOS) and poly(methylhydrosiloxane) (PMHS) were obtained from the chemical plant of Wuhan University (Wuhan, China). Trifluoroacetic acid (TFA) was purchased from Merck, Germany. The fused-silica fiber (120 *μ*m, o.d.) with protective polyimide coating was provided by Academy of Post and Telecommunication, Wuhan, China.

Custom standard mixtures (0.5 mg/mL for each) are C_8_–C_40_ *n*-alkanes (Accustandard), 16 polycyclic aromatic hydrocarbons (PAHs, Accustandard). The 16 PAHs (0.2 mg/mL for each) are naphthalene (C_10_H_8_), acenaphthylene (C_12_H_8_), acenaphthene (C_12_H_10_), fluorene (C_13_H_10_), phenanthrene (C_14_H_10_), anthracene (C_14_H_10_), fluoranthene (C_16_H_10_), pyrene (C_16_H_10_), benzo[a]anthracene (C_18_H_12_), Chrysene (C_18_H_12_), benzo[b]fluoranthene (C_20_H_12_), benzo[k]fluoranthene (C_20_H_12_), benzo[a]pyrene (C_20_H_12_), indeno[1,2,3-cd]pyrene (C_22_H_12_), dibenz[a,h]anthracene (C_22_H_14_), and benzo[g,h,i]perylene (C_22_H_12_). These compounds were used as external standards for compound identification and external quantification. 5-*α*-Androstane (Accustandard) was used as internal standards for quantification of terpanes and steranes.

### 2.3. Synthesis of MWNTs-TSO-OH

According to the literature [21], MWNTs were first refluxed in 2.6 M nitric acid for 45 h, oxidized by sulfuric acid (98%)/nitric acid (70%) for 24 h, and then stirred in SOCl_2_ at 70°C for 24 h. The product was thoroughly mixed with TSO-OH in a flask, heated to 75°C, and vigorously stirred under nitrogen protection for 48 h. The reaction mixture was dissolved in dichloromethane and filtered using a 0.2 *μ*m PTFE film. Ethanol was added to the filtrate and a black precipitate was obtained and proved to be MWNTs-TSO-OH.

### 2.4. Fiber Preparation

Prior to sol-gel coating, the 6 cm long fused-silica fiber was dipped in acetone for 3 h to remove the protective polyimide layer, in a 1 M NaOH solution for 1 h to expose the maximum number of silanol groups on the surface, cleaned with water, and dipped in 0.1 M HC1 solution for 30 min to neutralize the excess NaOH, cleaned again with water and air-dried at room temperature.

A sol solution was prepared by dissolving 90 mg of MWNTs-TSO-OH, 200 *μ*L of TEOS, 55 *μ*L of TSO-OH, 10 mg of PMHS, and 180 *μ*L of TFA (5% H_2_O) in 300 *μ*L of dichloromethane. The mixture was then mixed thoroughly by ultrasonic agitation for 5 min, centrifuged at 8000 rpm for 8 min, and the clear supernatant of the sol solution was transferred to another clean vial for fiber coating. The treated fiber was dipped vertically into the sol solution and held for 5 min, and then the fiber was drawn out of the sol solution, until a sol-gel coating was formed on the outer surface of the fiber end (about 1 cm). The coating process was repeated several times in the same sol solution until the desired thickness of the coating was obtained (70 *μ*m in this study). The fiber was irradiated under ultraviolet light for 30 min and then placed in a desiccator at room temperature for 24 h. The fiber was conditioned as follows: initially placed in a GC injection port at a 100°C with a gentle N2 flow for 1 h and then conditioned again at 220–340°C for 2 h.

### 2.5. Preparation of Working Standards and Samples

Compound identification and external quantification for the *n*-alkanes and PAHs were conducted through injections of two custom standard solutions, one containing C_8_–C_40_ *n*-alkanes and another containing a range of two to six ring PAHs. These standards were injected separately. C_8_–C_40_ standard solution was prepared in *n-*hexane at concentrations of 5, 10, 20, 50, 100, 200, 500, and 800 *μ*g/L. PAHs standard solution was prepared in *n-*hexane at concentrations of 2, 5, 10, 20, 50, 100, 200, and 500 *μ*g/L. For C_8_–C_40_ and PAHs standard solutions, 15 *μ*L of each was diluted with 15 mL of doubly deionized water, respectively. The final concentrations of C_8_–C_40_ working solution were 5, 10, 20, 50, 100, 200, 500, and 800 ng/L. The final concentrations of PAHs working solution were 2, 5, 10, 20, 50, 100, 200, and 500 ng/L. A standard solution of internal standard was prepared in *n-*hexane to yield 20 mg/mL of 5-*α*-Androstane. All solutions were stored at −20°C before use.

0.1 g of crude oil sample was dissolved in 50 mL of n-hexane and then dispersed by ultrasonic treatment for 5 min. After overnight stand, the solution was added into a funnel for filtering asphaltenes. The asphaltenes on the absorbent cotton were further rinsed by n-hexane for 3 times. All the filtrates were collected and concentrated to 2 mL by rotatable vacuum evaporator at 40°C. 10 *μ*L of concentrated sample was diluted with 15 mL of doubly deionized water.

### 2.6. DI-SPME Procedure

To obtain high extraction efficiency for the semivolatile compounds, DI-SPME was used. For each SPME analysis, 15 mL of standard solution or sample solution was placed into a 20 mL glass vial with 5 g of NaCl and a magnetic stir bar. The vial was tightly capped with a butyl rubber stopper wrapped with PTFE sealing tape and an aluminum cap. Then the stainless steel needle, where the fiber is housed, was pushed through the vial septum, and then the fiber was pushed out of the housing and immersed into the sample for 30 min at 70°C. After extraction, the fiber was removed from the sample vial and immediately inserted into the GC × GC injector port at 300°C with 5 min desorption time. Blank runs were performed before sampling to eliminate any carry-over of analytes from the previous extraction.

## 3. Results and Discussion

### 3.1. Characteristics of MWNTs-TSO-OH Fiber

#### 3.1.1. Thermal Stability and Lifespan of the Coating

**Figure 1:**
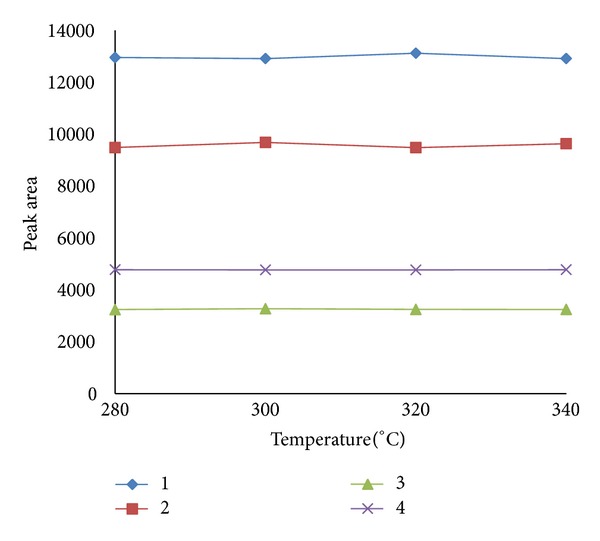
Thermal stability of MWNTs-TSO-OH fiber. Conditions: extraction time, 30 min; extraction temperature, 70°C; and desorption time, 5 min. Peaks: 1, *n*C_17_-alkane; 2, pristine; 3, naphthalene; and 4, 5-*α*-Androstane.

As an important parameter of SPME fiber, the thermal stability was investigated by performing extraction of *n*-alkanes and PAHs standard solutions after the fiber is being exposed at the GC injector port for 1 h at 280, 300, 320, and 340°C, respectively.  Figure 1 shows the peak areas of 4 representative compounds (*n*C_17_-alkane, pristine, naphthalene, and 5-*α*-Androstane) at different injector temperatures. It can be seen that the fiber exhibits excellent thermal stability up to 340°C without loss of extraction efficiency (peak area). Enhanced thermal stability allowed the use of higher injector temperatures for efficient desorption of semivolatile analytes, which contributes to the analysis of extended range of analytes including the high-boiling-point terpanes and steranes.

Under the high temperature along with long extraction time, the MWNTs-TSO-OH coating's extraction efficiency was monitored after it had been used for 50, 80, and 100 adsorption/desorption times. Due to the special nature of MWNTs and the strong chemical bonding provided by sol-gel technology, no obvious decline was observed after it had been used for 100 times.

#### 3.1.2. Preparation Reproducibility

Ten MWNTs-TSO-OH fibers (five prepared within a batch and five in different batches) with 70 *μ*m fiber thickness were used to evaluate the fibers' preparation reproducibility. The extractions of *n*-alkanes and PAHs standard solutions were performed in the same condition, and the peak areas of 4 representative compounds (*n*C_17_-alkane, pristine, naphthalene, and 5-*α*-Androstane) were selected to compare.  Table 1 shows that the relative standard deviation (RSD) of peak areas is less than 6% within a batch and 9% in different batches, respectively. Apparently, the sol-gel MWNTs-TSO-OH fibers have good reproducibility and are suitable for the accurate quantitative analysis of volatile and semivolatile compounds in crude oils.

### 3.2. Optimization of SPME Procedures

**Figure 2:**
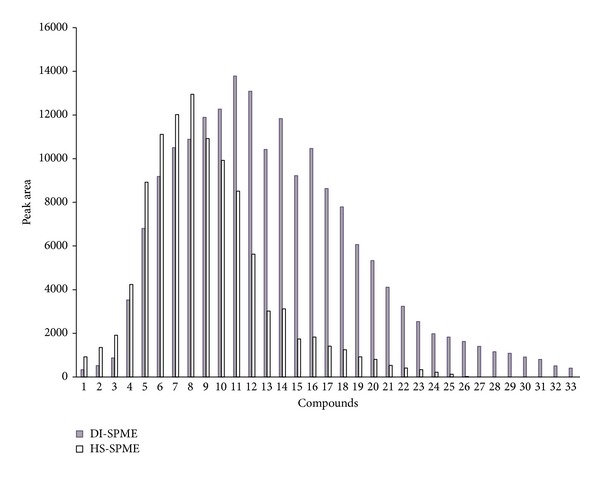
The extraction capability of the sol-gel coated MWNTs-TSO-OH fiber in DI-SPME mode and HS-SPME mode with the C_8_–C_40_ standard solution. Peaks: 1, n-octane; 2, n-nonane; 3, n-decane; 4, n-undecane; 5, n-dodecane; 6, n-tridecane; 7, n-tetradecane; 8, n-pentadecane; 9, n-hexadecane; 10, n-heptadecane; 11, n-octadecane; 12, n-nonadecane; 13, n-eicosane; 14, n-heneicosane; 15, n-docosane; 16, n-tricosane; 17, n-tetracosane; 18, n-pentacosane; 19, n-hexacosane; 20, n-heptacosane; 21, n-octacosane; 22, n-nonacosane; 23, n-triacontane; 24, n-hentriacontane; 25, n-dotriacontane; 26, n-tritriacontane; 27, n-tetratriacontane; 28, n-pentatriacontane; 29, n-hexatriacontane; 30, n-heptatriacontane; 31, n-octatriacontane; 32, n-nonatriacontane; and 33, n-tetracontane.

Figure 2 shows the extraction capability of the sol-gel coated MWNTs-TSO-OH fiber in DI-SPME mode and HS-SPME mode with the C_8_–C_40_ standard solution. It can be seen that HS-SPME exhibited high extraction efficiency for volatile compounds but failed within the range of C_17_–C_40_. On the contrary, DI-SPME allowed satisfactory results almost in the whole range of carbon number, especially for the semivolatile compounds. In order to obtain high sensitivity of some semivolatile biomarkers at trace level, DI-SPME mode was selected in this study.

**Figure 3:**
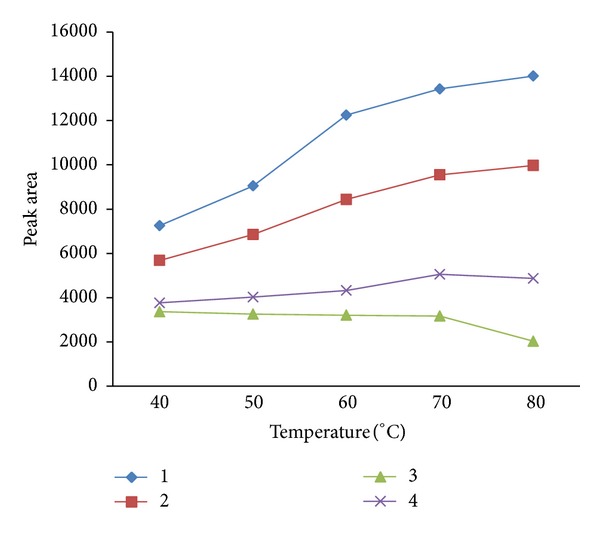
The extraction temperature profile for 4 representative compounds. Conditions: extraction time, 30 min; desorption time, 5 min; saturated out with NaCl; magnetic stirring. Peaks: 1, *n*C_17_-alkane; 2, pristine; 3, naphthalene; and 4, 5-*α*-Androstane.

Figure 3 shows the extraction temperature profile for 4 representative compounds (*n*C_17_-alkane, pristine, naphthalene, and 5-*α*-Androstane). Except for naphthalene, the extraction yield increased with an increase in temperature. In order to obtain high extraction efficiency for most volatile and semivolatile compounds, 70°C was selected as the optimum extraction temperature.

**Figure 4:**
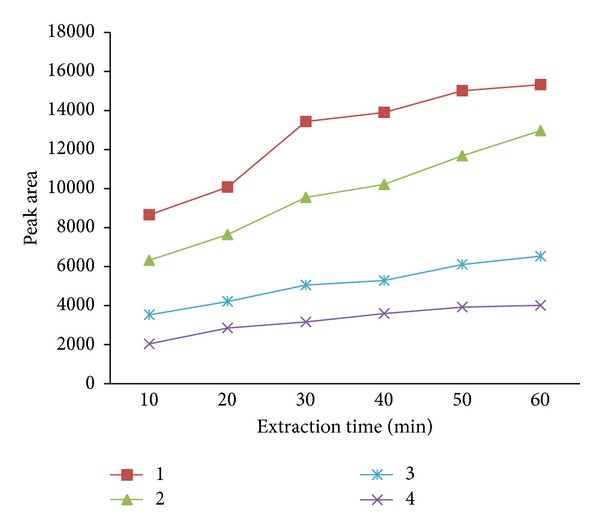
The extraction time profile for 4 representative compounds. Conditions: extraction temperature, 70°C; desorption time, 5 min; saturated out with NaCl; magnetic stirring. Peaks: 1, *n*C_17_-alkane; 2, pristine; 3, naphthalene; and 4, 5-*α*-Androstane.

The extraction times of 4 representative compounds (*n*C_17_-alkane, pristine, naphthalene, and 5-*α*-Androstane) were investigated from 10 to 60 min at 70°C (Figure 4). The responses of 4 compounds increased with the increase of extraction time, and the equilibrium could not be achieved. Due to the porous structure of the sol-gel MWNTs-TSO-OH coating, the analytes were extracted mainly by adsorption interaction, where a competitive process happened and the best extraction efficiency was not at the point of equilibrium [21]. Considering the sensitivity and time efficiency, 30 min was selected as the optimum extraction time in this study.

**Figure 5:**
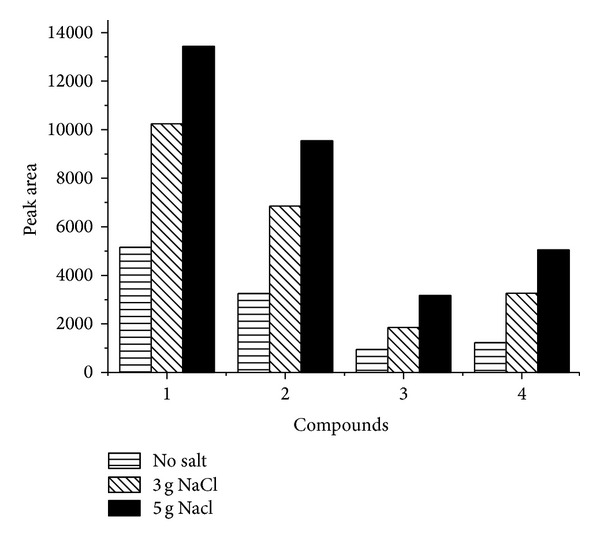
Effect of NaCl added on extraction. Conditions: extraction temperature, 70°C; extraction time, 30 min; desorption time, 5 min; magnetic stirring. Peaks: 1, *n*C_17_-alkane; 2, pristine; 3, naphthalene; and 4, 5-*α*-Androstane.

Salt effect is usually used to increase the extraction efficiency of analytes in SPME.  Figure 5 shows the effect of salt (NaCl) on the extraction of 4 representative compounds (*n*C_17_-alkane, pristine, naphthalene, and 5-*α*-Androstane). An obvious increase of peak area was observed with the increase of salt concentration. Apparently, increase of ion strength is beneficial to the transfer of analytes from solution to MWNTs-TSO-OH coating. Therefore, 5 g NaCl was added into each standard solution or sample solution in this study.

### 3.3. Characterization of Components in a Real Crude Oil Sample by DI-SPME-GC × GC/TOFMS

**Figure 6:**
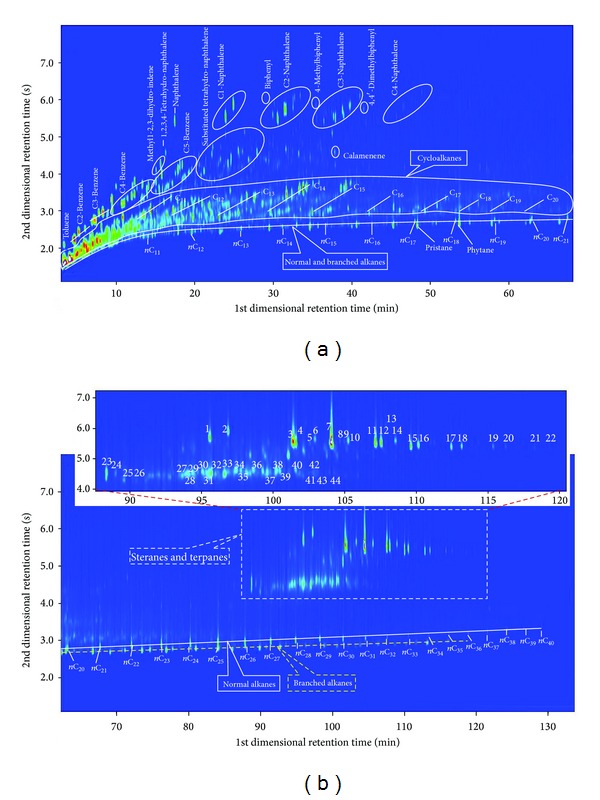
GC × GC/TOFMS total ion current chromatogram of a crude oil sample.

Analysis of a real crude oil sample from an oil field in China was performed by DI-SPME-GC × GC/TOFMS using the optimized conditions. The total ion current chromatogram is shown in Figure 6. Owing to the different interaction of compounds towards the stationary phase, the apparent group type separation can be observed. The 2D plane is divided into four separated zones according to the class of compounds: normal and branched alkanes, cycloalkanes, aromatic hydrocarbons, and biomarkers of crude oil such as steranes and terpanes. By combining the automated data processing of TOFMS software, the ordered GC × GC chromatogram, and the retention index database developed by our group, compounds in the oil sample were identified in detail. The detailed information and availability of the retention index database have been described in a previous publication [22].

#### 3.3.1. Paraffins and Naphthenes

The normal and branched alkanes are located at the bottom of the 2D plane, and branched alkanes tend to occupy the lower part with the number of carbon atom increasing. Additionally, some acyclic isoprenoids are also included in this region and clearly visible as resolved peaks. The cycloalkanes are located at upper part compared with saturated hydrocarbons. Within the cycloalkanes, individual compounds are clearly resolved, and apparent roof-tile effect is observed. Compounds regularly distribute along oblique lines according to the degree of branching and number of alkyl-substituents attached to the cyclic group, which are ultimately identified as the branched cyclopentane and cyclohexane isomers. Some important paraffins and naphthenes are marked in Figure 6 and listed in Table 2.

#### 3.3.2. Aromatic Hydrocarbons

It can be seen from Figure 6 that GC × GC completely separates the aromatic hydrocarbons from paraffins and naphthenes and shows all of them in the same chromatogram, which cannot be obtained in 1DGC analysis owing to the serious overlap of peaks and the lower amount of aromatic hydrocarbons relative to saturated hydrocarbons. Compared with paraffins and naphthenes, aromatic hydrocarbons are located at the upper part of the 2D plane because of their higher polarity. In the second dimension, compounds elute in proper order from mono-, di-, to triaromatic hydrocarbons. Isomers are grouped into the same band of the chromatogram and can be recognized owing to the roof-tile effect. The different groups are marked in Figure 6(a), and the identification of some important compounds is listed in Table 2.

#### 3.3.3. Biomarkers of Crude Oil

Polycyclic terpanes and steranes are the widely used biomarkers in petroleum geochemistry, owing to their general resistance to weathering, biodegradation, evaporation and other processes. They can be used by geologists to interpret the characteristics of petroleum source rocks when only oil samples are available. Additionally, they can also provide some important information on the organic matter in the source rock and environmental conditions during its deposition and burial, the thermal maturity experienced by rock or oil, the degree of biodegradation, and some aspects of source rock mineralogy and age [1]. But in high mature, over mature oil or source rock, they are usually difficult to be identified owing to their quite low content. In this study, the polycyclic terpanes and steranes are clearly resolved and identified using DI-SPME-GC × GC/TOFMS. In Figure 6(b), the blowup of the marked region shows the GC × GC separation of polycyclic terpanes and steranes. Compared with traditional 1DGC/MS methods, better separation is obtained and more trace components are identified. The identification of marked peaks is listed in Table 3. It can be seen from Figure 6(b) and Table 3 that abundant tri- and tetracyclic diterpanes appear in the crude oil, steranes show a low proportion to triterpanes, and C_29_ steranes predominate over C_27_ and C_28_ homologs.

### 3.4. Quantitative Calibration and Reproducibility

Based on the sol-gel MWNTs-TSO-OH coating, a series of experiments were performed to validate the DI-SPME-GC × GC/TOFMS analysis of volatile and semivolatile compounds in crude oils.  Table 4 summarizes the precision, limits of detection (LODs), and linearity ranges of the proposed method. The precision of the method was expressed as the relative standard deviation (RSD) and determined by analysis of standard solutions in five times. The values obtained were all below 8%, illustrating the good reproducibility of this method. Limit LODs, estimated on the basis of a signal-to-noise ratio of 3, ranged from 0.2 to 1.6 ng/L. The linearity was satisfactory, with the correlation coefficient (*r*) over 0.996. The linear range was from 10 to 500 ng/L.

## 4. Conclusions

A sol-gel MWNTs-TSO-OH SPME fiber was prepared and coupled to GC × GC/TOFMS for the analysis of the volatile and semivolatile organic compounds in crude oils. Compared with the commercial SPME fibers, MWNTs-TSO-OH fiber exhibited high sensitivity, thermal stability, and long lifespan. The proposed method provided a simple, efficient, and sensitive tool to simultaneously analyze the normal and branched alkanes, cycloalkanes, aromatic hydrocarbons, and biomarkers of crude oil such as steranes and terpanes. Furthermore, the method showed good linearity (*r* > 0.999), precision (RSD < 8%), and detection limits ranging from 0.2 to 1.6 ng/L.

## Figures and Tables

**Table 1:** Preparation reproducibility of MWNTs-TSO-OH fibers with 70 *μ*m thickness.

Compounds	RSD^a^ (%)	
	Fibers within a batch (*n* = 5)	Fibers in different batches (*n* = 5)
*n*C_17_ *-*alkane	1.21	2.86
Pristine	3.50	5.18
Naphthalene	5.27	8.12
5-*α*-Androstane	4.63	8.39

^a^RSD: relative standard deviation, calculated according to the peak areas.

**Table 2:** Identification of some important paraffins, naphthens, and aromatic hydrocarbons.

^1^ *t*_*R*_	^2^ *t*_*R*_	Compounds	Family	^1^ *t*_*R*_	^2^ *t*_*R*_	Compounds	Family
3.17	2.06	Toluene	1A	42.75	6.42	4-Methyldibenzofuran	2A
4.50	2.28	1,3-Dimethyl benzene	1A	43.33	6.06	Ethylbiphenyl	2A
4.50	2.31	1,2-Dimethyl benzene	1A	43.42	6.34	2-Methyldibenzofuran	2A
4.58	2.34	Ethyl benzene	1A	43.92	6.69	3-Methyldibenzofuran	2A
4.75	2.35	1,4-Dimethyl benzene	1A	44.42	5.58	1,3,5,7-Tetramethylnaphthalene	2A
4.92	1.79	1,2,3-Trimethyl cyclohexane	PN	45.58	5.55	1,3,6,7-Tetramethylnaphthalene	2A
5.08	1.85	1-Ethyl-4-methyl cyclohexane	PN	46.42	5.82	1,4,6,7-Tetramethylnaphthalene	2A
5.17	1.96	1-Propyl cyclohexane	PN	46.58	5.86	1,2,5,7-Tetramethylnaphthalene	2A
5.33	1.84	1,1,3,5-Tetramethylcyclohexane	PN	46.75	6.43	2-Methylfluorene	2A
5.92	2.64	1-Methylethyl benzene	1A	46.92	5.60	2,3,6,7-Tetramethylnaphthalene	2A
6.00	2.12	1-Ethyl-3-methyl cyclohexane	PN	47.17	6.69	1-Methylfluorene	2A
6.08	2.08	1-Ethyl-2-methyl cyclohexane	PN	47.50	5.92	1,2,6,7-Tetramethylnaphthalene	2A
6.50	1.91	1,2,3,5-Tetramethylcyclohexane	PN	47.67	6.02	1,2,3,7-Tetramethylnaphthalene	2A
6.83	2.80	Propyl benzene	1A	47.75	6.99	3-Methylfluorene	2A
7.08	2.79	1-Ethyl-4-methyl benzene	1A	47.92	6.00	1,2,3,6-Tetramethylnaphthalene	2A
7.08	2.87	1-Ethyl-3-methyl benzene	1A	47.92	2.74	n-Heptadecane	PN
7.17	2.90	1,2,3-Trimethyl benzene	1A	48.25	2.61	Pristane	PN
7.33	2.85	1,2,4-Trimethyl benzene	1A	48.75	6.22	1,2,5,6-Tetramethylnaphthalene	2A
7.67	2.03	1-Ethyl-1,3-dimethylcyclohexane	PN	49.42	7.92	Dibenzothiophene	2A
7.75	2.94	1-Ethyl-2-methyl benzene	1A	50.83	7.90	Phenanthrene	3A
7.83	2.09	1-Tethyl-2-propyl cyclohexane	PN	52.67	6.21	2-Ethylfluorene	2A
7.83	2.04	1,1,3,4-Tetramethyl cyclopentane	PN	52.92	6.37	3,6-Dimethylfluorene	2A
8.00	2.04	1,1,3,3,5-Pentamethylcyclohexane	PN	53.08	2.75	n-Octadecane	PN
8.08	2.05	1,5-Diethyl-2,3-dimethylcyclohexane	PN	53.25	6.43	1-Ethylfluorene	2A
8.33	1.95	n-Decane	PN	53.50	6.54	2,6-Dimethylfluorene	2A
8.33	2.08	Ethyl propyl cyclopentane	PN	53.83	2.69	Phytane	PN
8.50	2.10	1,1,3,3,5-Pentamethylcyclohexane	PN	54.50	7.50	4-Methyldibenzothiophene	2A
9.62	3.02	1-Ethyl-2-methyl benzene	1A	55.00	6.77	2,7-Dimethylfluorene	2A
10.08	3.11	1-Methyl-2-(1-methylethyl) benzene	1A	55.58	7.49	2-Methyldibenzothiophene	2A
10.67	3.18	1-Methyl-3-propyl benzene	1A	56.17	6.22	1,2,4,6,7-Pentamethylnaphthalene	2A
10.83	3.21	2-Methylpropyl benzene	1A	56.58	7.44	3-Methylphenanthrene	3A
11.08	3.41	1-Methyl-2-(1-methylethyl) benzene	1A	56.67	7.95	1-Methyldibenzothiophene	2A
11.17	3.12	2,2-Dimethylpropyl benzene	1A	56.83	7.54	2-Methylphenanthrene	3A
12.00	3.37	Adamantane	PN	57.75	7.81	9-Methylphenanthrene	3A
12.00	3.69	1-Butenyl benzene	1A	58.00	7.82	1-Methylphenanthrene	3A
12.83	3.17	1-Methyladamantane	PN	58.08	2.78	n-Nonadecane	PN
13.00	2.20	n-Undecane	PN	59.33	5.98	4-Ethyl dibenzothiophene	2A
13.08	3.28	1-Methyl-4-(2-methylpropyl)benzene	1A	59.42	7.26	4,6-Dimetyl dibenzothiophene	2A
13.08	3.37	1-Methylbutylbenzene	1A	60.17	7.09	2,4-Dimetyl dibenzothiophene	2A
13.25	3.41	1-Ethyl-4-(1-methylethyl)benzene	1A	60.42	7.74	2-Phenylnaphthalene	3A
13.58	2.97	1,3-Dimethyl adamantane	PN	60.50	7.23	2,6-Dimetyl dibenzothiophene	2A
18.50	2.43	n-Dodecane	PN	61.17	7.17	3-Ethylphenanthrene	3A
23.92	5.50	2-Methylnaphthalene	2A	61.92	7.25	2-Ethylphenanthrene	3A
24.75	2.54	n-Tridecane	PN	62.00	7.06	9-Ethylphenanthrene	3A
24.83	5.78	1-Methylnaphthalene	2A	62.08	7.60	3,6-Dimethylphenanthrene	3A
29.00	6.06	1,1′-Biphenyl	2A	62.33	6.30	3,6-Dimetyl dibenzothiophene	2A
29.83	5.60	2-Ethylnaphthalene	2A	62.33	7.15	3,5-Dimethylphenanthrene	3A
30.00	5.92	1-Ethylnaphthalene	2A	62.50	7.26	2,7-Dimethylphenanthrene	3A
30.33	5.73	Diphenylmethane	2A	62.58	3.07	3,7-Dimetyl dibenzothiophene	2A
30.42	5.52	2,6-Dimethylnaphthalene	2A	62.83	2.80	n-Eicosane	PN
30.83	2.61	n-Tetradecane	PN	63.17	7.36	2,10-Dimethylphenanthrene	3A
31.33	5.76	1,7-Dimethylnaphthalene	2A	63.42	7.41	2,5-Dimethylphenanthrene	3A
31.50	5.82	1,3-Dimethylnaphthalene	2A	63.67	7.54	1,7-Dimethylphenanthrene	3A
32.25	6.22	2-Methylbiphenyl	2A	64.00	7.44	2,3-Dimethylphenanthrene	3A
32.42	5.79	1,4-Dimethylnaphthalene	2A	64.17	7.71	1,9-Dimethylphenanthrene	3A
32.58	6.08	1,5-Dimethylnaphthalene	2A	64.75	7.73	1,8-Dimethylphenanthrene	3A
33.33	6.11	1,2-Dimethylnaphthalene	2A	65.50	6.91	1,2-Dimethylphenanthrene	3A
35.33	5.89	3-Methylbiphenyl	2A	65.58	7.41	6-Methyl-2-phenylnaphthalene	3A
35.83	5.92	4-Methylbiphenyl	2A	65.75	7.22	7-Methyl-2-phenylnaphthalene	3A
36.00	5.60	2,3′-Dimethylbiphenyl	2A	66.00	5.86	1-Methyl-7-phenylnaphthalene	3A
36.33	5.44	2-Methyl-7-ethylnaphthalene	2A	66.33	2.70	Pyrene	4A
36.42	5.60	2,5-Dimethylbiphenyl	2A	66.42	7.04	1-Methyl-3-phenylnaphthalene	3A
36.75	2.66	n-Pentadecane	PN	67.33	2.83	n-Heneicosane	PN
36.75	5.66	2,4-Dimethylbiphenyl	2A	68.17	7.01	Trimethylphenanthrene	3A
36.75	6.58	Dibenzofuran	2A	71.67	2.88	n-Docosane	PN
37.33	5.66	1-Methyl-7-ethylnaphthalene	2A	75.83	2.90	n-Tricosane	PN
37.58	5.55	1,3,7-Trimethylnaphthalene	2A	79.83	2.91	n-Tetracosane	PN
37.83	5.50	1,3,6-Trimethylnaphthalene	2A	83.75	2.94	n-Pentacosane	PN
37.92	6.13	2,3-Dimethylbiphenyl	2A	87.42	2.98	n-Hexacosane	PN
38.67	5.73	1,3,5-Trimethylnaphthalene	2A	91.00	2.99	n-Heptacosane	PN
38.83	5.60	2,3,6-Trimethylnaphthalene	2A	94.50	3.02	n-Octacosane	PN
39.75	5.89	1,2,7-Trimethylnaphthalene	2A	97.83	3.06	n-Nonacosane	PN
39.92	5.26	1,2,6-Trimethylnaphthalene	2A	101.08	3.06	n-Triacontane	PN
40.50	6.14	1,2,4-Trimethylnaphthalene	2A	104.17	3.07	n-Hentriacontane	PN
40.50	6.80	Fluorene	2A	107.17	3.10	n-Dotriacontane	PN
40.58	5.82	3-Ethylbiphenyl	2A	110.17	3.14	n-Tritriacontane	PN
40.92	6.11	1,2,5-Trimethylnaphthalene	2A	113.00	3.15	n-Tetratriacontane	PN
41.42	5.74	3,3′-Dimethylbiphenyl	2A	115.75	3.22	n-Pentatriacontane	PN
41.83	6.29	1,2,3-Trimethylnaphthalene	2A	118.50	3.18	n-Hexatriacontane	PN
41.92	5.81	3,4′-Dimethylbiphenyl	2A	121.08	3.25	n-Heptatriacontane	PN
42.25	5.68	4,4′-Dimethylbiphenyl	2A	124.00	3.20	n-Octatriacontane	PN
42.42	2.69	n-Hexadecane	PN	126.17	3.30	n-Nonatriacontane	PN

PN: paraffins and naphthens; 1A: monoaromatic hydrocarbon; 2A: diaromatic hydrocarbon; 3A: triaromatic hydrocarbon; and 4A: tetra-aromatic hydrocarbon.

**Table 3:** Identification of polycyclic terpanes and steranes marked in Figure 6.

No.	^1^ *t*_*R*_	^2^ *t*_*R*_	Compounds	No.	^1^ *t*_*R*_	^2^ *t*_*R*_	Compounds
1	95.58	5.71	C_27_18*α*-trisnorhopane (Ts)	23	88.33	4.37	C_27_13*β*,17*α*-20S-diacholestane
2	96.83	5.92	C_27_17*α*-trisnorhopane (Tm)	24	89.08	4.30	C_27_13*β*,17*α*-20R-diacholestane
3	101.42	5.57	C_29_17*α*,21*β*-21-ethylhopane	25	89.62	4.32	C_27_13*α*,17*β*-20S-diacholestane
4	102.08	5.31	C_29_18*α*-30-norneohopane	26	91.00	4.51	C_27_13*α*,17*β*-20R-diacholestane
5	102.58	5.55	C_30_17*α*-diahopane	27	93.67	4.48	C_27_5*α*,14*α*,17*α*-20S-cholestane
6	102.92	5.65	C_29_17*β*,21*α*-21-ethylmoretane	28	94.08	4.51	C_27_5*α*,14*β*,17*β*-20R-cholestane
7	104.08	5.58	C_30_17*α*,21*β*-21-isopropylhopane	29	94.33	4.56	C_27_5*α*,14*β*,17*β*-20S-cholestane
8	104.67	5.58	C_30_pentacyclic triterpane	30	95.00	4.61	C_27_5*α*,14*α*,17*α*-20R-cholestane
9	104.92	5.58	C_29_17*β*,21*β*-21-ethylhopane	31	95.08	4.59	C_29_13*β*,17*α*-20R-24-ethyldiacholestane
10	105.25	5.62	C_30_17*β*,21*α*-21-isopropylhopane	32	95.42	4.56	C_29_13*α*,17*β*-20S-24-ethyldiacholestane
11	107.17	5.55	C_31_17*α*,21*β*-22S-21-isobutylhopane	33	96.83	4.64	C_28_5*α*,14*α*,17*α*-20S-24-methylcholestane
12	107.50	5.55	C_31_17*α*,21*β*-22R-21-isobutylhopane	34	97.42	4.67	C_28_5*α*,14*β*,17*β*-20R-24-methylcholestane
13	107.83	6.13	Gammacerane	35	97.75	4.64	C_28_5*α*,14*β*,17*β*-20S-24-methylcholestane
14	108.50	5.60	C_31_17*β*,21*α*-21-isobutylmoretane	36	98.58	4.66	C_28_5*α*,14*α*,17*α*-20R-24-methylcholestane
15	109.58	5.44	C_32_17*α*,21*β*-22S-21-isopentylhopane	37	99.58	4.59	C_29_5*α*,14*α*,17*α*-20S-24-ethylcholestane
16	110.17	5.46	C_32_17*α*,21*β*-22R-21-isopentylhopane	38	100.08	4.58	C_29_5*α*,14*β*,17*β*-20R-24-ethylcholestane
17	112.42	5.42	C_33_17*α*,21*β*-22S-21-isohexylhopane	39	100.50	4.62	C_29_5*α*,14*β*,17*β*-20S-24-ethylcholestane
18	113.17	5.42	C_33_17*α*,21*β*-22R-21-isohexylhopane	40	101.50	4.66	C_29_5*α*,14*α*,17*α*-20R-24-ethylcholestane
19	115.33	5.42	C_34_17*α*,21*β*-22S-21-isoheptylhopane	41	102.50	4.58	C_30_5*α*,14*α*,17*α*-20S-4-methyl-24-ethylcholestane
20	116.25	5.42	C_34_17*α*,21*β*-22R-21-isoheptylhopane	42	102.75	4.59	C_30_5*α*,14*β*,17*β*-20R-4-methyl-24-ethylcholestane
21	118.25	5.38	C_35_17*α*,21*β*-22S-21-isooctylhopane	43	102.83	4.58	C_30_5*α*,14*β*,17*β*-20S-4-methyl-24-ethylcholestane
22	119.33	5.41	C_35_17*α*,21*β*-22R-21-isooctylhopane	44	103.92	4.56	C_30_5*α*,14*α*,17*α*-20R-4-methyl-24-ethylcholestane

**Table 4:** Precisions (RSD), limits of detection (LODs), and linear ranges of the proposed method.

Compounds	RSD^a^ (%) (*n* = 5)	LODs^b^ (ng/L)	Linear range (ng/L)	Regression equation	*r*
*n*C_17_-alkane	3.6	0.2	10–800	*y* = 0.0018 + 0.0132*x*	0.9998
Pristine	5.1	0.5	10–800	*y* = 0.0105 + 0.0267*x*	0.9993
Naphthalene	7.8	1.6	10–500	*y* = 0.0237 + 0.0087*x*	0.9965
5-*α*-Androstane	4.6	0.8	10–500	*y* = 0.0138 + 0.0032*x*	0.9971

^a^The concentration of the standard solution was 50 ng/L for each compound.

^b^LODs were estimated on the basis of a signal-to-noise ratio of 3.
